# Survival Rate in Lung, Liver, Heart and Pancreas Transplant Recipients in Iran: A Registry-Based Study

**Published:** 2013-08-01

**Authors:** Fatemeh Ghaemi, Farahnaz Ghaemi, M. Zamyad

**Affiliations:** 1 *Ministry of Health and Medical Education, Department of Transplantation and Specific Diseases, *; 2 *Islamic Azad University*

**Keywords:** Lung transplantation, liver transplantation, heart transplantation, pancreas transplantation, recipient survival rate, ischemic time

## Abstract

Background: The main purpose of organ transplantation is to prolong and maintain a quality life for patients with organ dysfunction.

Objective: We tried to evaluate short-term survival rates in lung, liver, heart and pancreas recipients.

Methods: This longitudinal study was based on the data of national registry of recipients in Ministry of Health and Medical Education (MOHME), Iran. Survival rate after 1 year of transplantation, recipients’ age, gender, ischemic time as well as the number of transplantation units, OP units and identification units were collected from the database for all transplantation done between 2010 and 2011.

Results: 407 (223 female, and 184 male) patients were enrolled in the study. 13 (3.2%) patients received lung, 299 (73.5%) liver, 85 (20.9%) heart and 10 (2.5%) received pancreas. Within 1 year of transplantation, 61.5% of lung recipients, 88.3% of liver recipients, 72.9% of heart recipients and 80% of pancreas recipients (overall 85.3%) were functioning.

Conclusion: Given the short history of transplantation in Iran, we have achieved great success.

## INTRODUCTION

Nowadays, patients with untreatable end-stage organ disease are considered for organ transplantation [1-3]. The major constraint on meeting the demand for transplantation is the availability of donated (cadaver) organs [4, 5]. Introduction of new immunosuppressants over the past 20 years along with improvements achieved in infection prophylaxis strategies, have resulted in a remarkable improvement in both patients and grafts survival rates. These factors have changed organ transplantation into the treatment of choice for patients with end-stage organ disease [6, 7]. However, other factors such as age and gender of recipients and donors, background diseases and cold ischemic time can change survival rate [8-10]. Therefore, we conducted this study to assess the short-term survival rate in lung, liver, heart and pancreas transplant recipients based on data of the Ministry of Health and Medical Education (MOHME) registry, Iran.

## PATIENTS AND METHODS

This longitudinal study was based on the data retrieved from Iranian MOHME registry. All requests for lung, liver, heart and pancreas are registered in MOHME transplantation database by the staff in the transplantation wards of hospitals in Iran. The recipients are followed after transplantation in two ways: first, the patients are followed in the same hospital where transplantation took place; second, the staff members of the transplantation department in the Ministry of Health follow them up by phone.

The staff members of the transplantation wards in all hospitals are required to enter the results of follow-ups into the said database. However, owing to some limitations, not all the required data are entered into the database. The only piece of information that is received for all cases is their death/survival.

The total number of people having organ transplantation in Iran in 2010 was 2454; of these, 407 were studied in the present research (transplantations in 2010 and 2011); the rest of patients have had kidney transplantation which is not the focus of this study. Of 407 recipients, 223 (54.8%) were female and 184 (45.2%) were male.

All recipients receiving lung, liver, heart and pancreas were followed for one year. In all cases of transplantation, donors and recipients did not know each other; the only exception was in case of living donors of liver where both the recipient and donor were related.

Then relevant data on recipients including their number, age, gender, and cold ischemic time, survival and death within one year after transplantation, donors’ characteristics (*i.e.*, deceased or live) as well as the organ transplantation center were collected and tabulated.

## RESULTS

Most of the liver transplant donors were deceased. Table 1 shows data on active centers in Iran where lung, liver, heart and pancreas are transplanted. Table 2 demonstrates the total number of recipients as well as their gender, age and cold ischemic time. Table 3 depicts the distribution of transplants stratified by type of donor. Table 4 reveals recipients’ survival within one year of transplantation stratified by type of donor.

**Table 1:** Active organ transplantation centers in Iran

Organ	No. of transplantation units	No. of organ procurement units	No. of identification units
Lung	2	14	30
Liver	5		
Heart	4		
Pancreas	1		
Total	12	14	30

**Table 2:** Recipients’ data

Organ	Total No. of recipients	No. of female recipients	Mean age (yrs)	Cold ischemic time (h)
Lung	13 (3.2%)	4 (31%)	34.3	2–4
Liver	299 (73.5%)	191 (63.9%)	33.2	3–8
Heart	85 (20.9%)	23 (27%)	33.4	2–4
Pancreas	10 (2.5%)	5 (50%)	29.8	3–8
Total	407	223 (54.8%)		

**Table 3:** Distribution of transplants stratified by type of donor

No.	Organ	Total No. of Recipients	No. of Recipients from Deceased Donors	No. of Recipients from Live Donors
1	Lung	13 (3.20%)	13 (3.20%)	0
2	Liver	299 (73.46%)	252 (84.28%)	47 (15.72%)
3	Heart	85 (20.88%)	85 (20.88%)	0
4	Pancreas	10 (2.46%)	10 (2.46%)	0
Total		407	371	36

**Table 4:** Recipients’ survival within one year of transplantation

Organ	Total No. of recipients	No. of active recipients from deceased donors	No. of inactive recipients from deceased donors	No. of active recipients from live donors	No. of inactive recipients from live donors
Lung	13	8 (62%)	5 (38%)	0 (0%)	0 (0%)
Liver	299	222 (74.3%)	30 (10.0%)	42 (14.1%)	5 (1.7%)
Heart	85	62 (73%)	23 (27%)	0 (0%)	0 (0%)
Pancreas	10	8 (80%)	2 (20%)	0 (0%)	0 (0%)
Total	407	300	60	42	5

## DISCUSSION

Over the past decade, with improvements in organ transplantation, the number of indications for organ transplantation has continuously grown and the need for organ donation has steadily increased. Our results showed that transplanted organs were functioning in majority of recipients (85.3%) within one year of transplantation. Because there has been only a short period (10 years) since the establishment of transplantation law from deceased donors in Iran, this survival rate can be considered a success. This significant success could not have been achieved without the painstaking efforts of our dedicated physicians and staff members as well as the recipient, donor and allocation coordinators. We owe a lot to the hardworking staff members in transplantation units, identification units and OPUs. The support and cooperation we have received from the authorities of hospitals, universities and the Ministry of Health need appreciation too.

The small number of failures in our series may be attributed to lack of facilities and trained personnel in other universities. We hope to achieve better results in near future by increasing the number of transplantation wards, OPUs, and identification units in hospitals all over the country. An increase in the number of well-equipped centers will hopefully reduce ischemic time. Training people on how to prevent organ failure can also diminish the demand for organ transplantation. Besides, good patient follow-up after transplantation may decrease the complications and increase the survival rate [11, 12].
